# Interferon-γ increases neuronal death in response to amyloid-β_1-42_

**DOI:** 10.1186/1742-2094-3-7

**Published:** 2006-03-28

**Authors:** Clive Bate, Sarah Kempster, Victoria Last, Alun Williams

**Affiliations:** 1 Department of Pathology and Infectious Diseases, Royal Veterinary College, Hawkshead Lane, North Mymms, Herts, AL9 7TA, UK

## Abstract

**Background:**

Alzheimer's disease is a neurodegenerative disorder characterized by a progressive cognitive impairment, the consequence of neuronal dysfunction and ultimately the death of neurons. The amyloid hypothesis proposes that neuronal damage results from the accumulation of insoluble, hydrophobic, fibrillar peptides such as amyloid-β_1-42_. These peptides activate enzymes resulting in a cascade of second messengers including prostaglandins and platelet-activating factor. Apoptosis of neurons is thought to follow as a consequence of the uncontrolled release of second messengers. Biochemical, histopathological and genetic studies suggest that pro-inflammatory cytokines play a role in neurodegeneration during Alzheimer's disease. In the current study we examined the effects of interferon (IFN)-γ, tumour necrosis factor (TNF)α, interleukin (IL)-1β and IL-6 on neurons.

**Methods:**

Primary murine cortical or cerebellar neurons, or human SH-SY5Y neuroblastoma cells, were grown in vitro. Neurons were treated with cytokines prior to incubation with different neuronal insults. Cell survival, caspase-3 activity (a measure of apoptosis) and prostaglandin production were measured. Immunoblots were used to determine the effects of cytokines on the levels of cytoplasmic phospholipase A_2 _or phospholipase C γ-1.

**Results:**

While none of the cytokines tested were directly neurotoxic, pre-treatment with IFN-γ sensitised neurons to the toxic effects of amyloid-β_1-42 _or HuPrP82-146 (a neurotoxic peptide found in prion diseases). The effects of IFN-γ were seen on cortical and cerebellar neurons, and on SH-SY5Y neuroblastoma cells. However, pre-treatment with IFN-γ did not affect the sensitivity to neurons treated with staurosporine or hydrogen peroxide. Pre-treatment with IFN-γ increased the levels of cytoplasmic phospholipase A_2 _in SH-SY5Y cells and increased prostaglandin E_2 _production in response to amyloid-β_1-42_.

**Conclusion:**

Treatment of neuronal cells with IFN-γ increased neuronal death in response to amyloid-β_1-42 _or HuPrP82-146. IFN-γ increased the levels of cytoplasmic phospholipase A_2 _in cultured neuronal cells and increased expression of cytoplasmic phospholipase A_2 _was associated with increased production of prostaglandin E_2 _in response to amyloid-β_1-42 _or HuPrP82-146. Such observations suggest that IFN-γ produced within the brain may increase neuronal loss in Alzheimer's disease.

## Background

Alzheimer's disease (AD) is a neurodegenerative disorder characterized by progressive cognitive impairment as a consequence of neuronal dysfunction and loss. The amyloid hypothesis maintains that the neuronal dysfunction and death that give rise to the clinical symptoms of AD are caused by the accumulation of fibrils consisting of amyloid-β peptides [1]. These peptides are formed following the cleavage of the amyloid precursor protein by γ-secretases [2], and depositions of amyloid-β peptides are a component of the senile plaques found in diseased brains [3]. The neuronal loss that occurs in AD has been modelled *in vitro *by incubating neurons with specific peptides derived from the amyloid-β protein [4]. The neuronal injury induced by these peptides includes characteristics of apoptosis such as chromatin condensation and DNA fragmentation [5].

In AD, amyloid deposits containing fibrillar amyloid-β peptides frequently co-localise with inflammatory cells strongly suggesting that the deposits of amyloid-β stimulate a chronic inflammatory process [6]. Genetic studies have identified polymorphisms in the genes of some inflammatory cytokines as risk factors for AD [7] suggesting that cytokine production within the brain may influence neuropathogenesis. While the effects of cytokines on astroglial cells within the brain are well reported, less is known about the direct effects of individual cytokines on neurons. In the current study we report that pre-treatment with interferon (IFN)-γ significantly increased the sensitivity of neurons to the toxic effects of amyloid-β_1-42_. The increased sensitivity of IFN-γ treated neurons to amyloid-β_1-42 _correlated with increased expression of cytoplasmic phospholipase A_2 _(cPLA_2_) in neuroblastoma cells and increased prostaglandin production in response to exogenous amyloid-β_1-42_. These results are consistent with prior observations that uncontrolled activation the cPLA_2_/cyclo-oxygenase (COX) pathway by amyloid-β_1-42 _leads to neuronal death [8].

## Methods

### Cell lines

The human neuroblastoma cell line SH-SY5Y was grown in RPMI-1640 medium supplemented with 2 mM glutamine, standard antibiotics (100 U/ml Penicillin, 100 μg/ml Streptomycin) and 2% fetal calf serum (FCS). For toxicity studies cells were seeded at 3 × 10^4 ^cells per well in 48 well plates, treated with cytokines and allowed to adhere overnight before use. After 24 hours, different concentrations of peptides, staurosporine or hydrogen peroxide were added. Cell viability and/or prostaglandin E_2 _content were determined after a further 24 hours.

### Primary neuronal cultures

Primary cortical neurons were prepared from embryonic day 15.5 mice as previously described [9]. Neuronal progenitors were seeded at 500,000 cells per well in 48 well plates in RPMI-1640 supplemented with 2 mM glutamine, standard antibiotics and 10% FCS. After 2 hours, cultures were washed and subsequently grown in neurobasal medium containing 2 mM glutamine and B27 components (Invitrogen, Paisley, UK). Primary cerebellar neurons were prepared from the brains from newborn mice pups following dissection of the cerebellum, removal of the meninges and cell dissociation as previously described [9]. Neuronal progenitors were plated in 10% FCS for 2 hours, and then grown in neurobasal medium containing glutamine and B27. In both these neuronal cultures, medium was supplemented with 5 mM L-leucine methyl ester to reduce the numbers of contaminating microglial cells. After 7 days, cultures were treated with cytokines for 24 hours before the addition of neurotoxins/peptides. Caspase-3 activity was measured 24 hours after the addition of neurotoxins using a flourometric immunosorbent enzyme assay kit as per the manufacturer's instructions (Roche Diagnostics, Lewes, UK). Results are expressed as fluorescent units which are proportional to caspase-3 activity. For toxicity assays medium was replaced 48 hours after the addition of neurotoxins/peptides and cell viability was determined after another 48 hours (4 days after the addition of neurotoxins/peptides).

### Peptides

A peptide corresponding to amino acids 1 to 42 of the amyloid-β protein (amyloid-β_1-42_) and a control peptide (amyloid-β_42-1_) were obtained from Bachem (St Helens, UK). Peptides containing amino acid residues 82 to 146 of the human PrP protein (HuPrP82-146) corresponding to a PrP fragment found in certain prion-infected human brains [10], a control peptide containing the same amino acids in a scrambled order (HuPrP82-146scrambled) were a gift from Professor Mario Salmona (Mario Negri Institute, Milan).

### Cell viability assays

To determine cell survival, cultures were treated with WST-1 (Roche Diagnostics Ltd, Lewes, UK) for 3 hours and optical density was read on a spectrophotometer at a wavelength of 450 nm. WST-1 is cleaved to formazan by mitochondrial dehydrogenases and the amount of dye formed correlates to the number of metabolically active cells. Percentage cell survival in cultures was calculated by reference to untreated cells incubated with WST-1 (100%).

### Cellular lysates

SH-SY5Y neuroblastoma cells were lysed in an extraction buffer containing 10 mM Tris-HCl, pH 7.8, 100 mM sodium chloride, 10 mM EDTA, 0.5% Nonidet P-40, 0.5% sodium deoxycholate and 2 mM phenylmethylsulphonylflouride at 1 × 10^6 ^cells per ml. Protein content was determined using a BCA kit (Pierce, Cramlington UK) and protein concentrations standardised. 20 μl samples were analysed via PAGE or blotted onto a PVDF membrane. Where appropriate, dilutions of lysates were made prior to blotting. Blots were probed with monoclonal antibodies (mabs) to cPLA_2 _or phospholipase C (PLC)γ-1 (Upstate, Milton Keynes, UK) and developed with an anti-mouse IgG-alkaline phosphatase conjugate followed by BCIP/NBT (Sigma).

### Prostaglandin E_2 _assay

Analysis of total prostaglandin E_2 _levels was performed using an enzyme-immunoassay kit Amersham Biotech (Amersham, UK).

### Drugs

Recombinant murine TNFα, IL-6, IL-1β, IFN-γ were supplied from (R&D systems, Abingdon, UK). Human IFN- was obtained from (Sigma, Poole, UK).

### Statistical analysis

Comparison of treatment effects were carried out using one and two way analysis of variance techniques as appropriate. *Post hoc *comparisons of means were performed as necessary.

## Results

### Pre-treatment with IFN-γ reduces the survival of cortical neurons incubated with amyloid-β_1-42_

**Figure 1:**
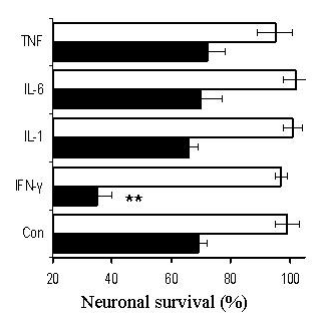
**Pre-treatment with IFN-γ reduces the survival of neurons incubated with amyloid-β_1-42_**. The survival of primary cortical neurons pre-treated with 1 ng/ml TNFα, IL-1β, IL-6 or IFN-γ prior to the addition of 10 μm amyloid-β_1-42 _(shaded bars) or 10 μm amyloid-β_42-1 _(open bars). Values shown are the mean percentage cell survival from triplicate experiments repeated 3 times (n = 9), ± standard deviation (SD). ** = Neuronal survival significantly less than untreated neurons incubated with amyloid-β_1-42 _(p < 0.05).

**Figure 2:**
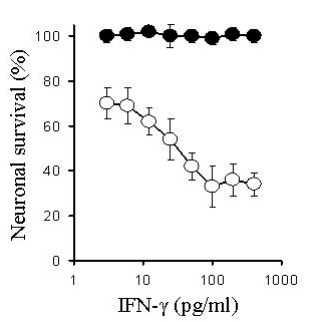
**The IFN-γ-induced sensitization of neurons to amyloid-β_1-42 _is dose-dependent**. The survival of primary cortical neurons pre-treated with different concentrations of IFN-γ prior to the addition of 10 μm amyloid-β_1-42 _(○) or 10 μm amyloid-β_42-1 _(●). Values shown are the mean percentage cell survival from triplicate experiments repeated 3 times (n = 9), ± SD.

Preliminary studies examined the effects of varying concentrations of murine cytokines (0.01 to 10 ng/ml) on the survival of primary murine cortical neurons. We were unable to detect any significant reduction in the survival of neurons following culture with any of the following recombinant murine cytokines; TNF-α, IL-1β, IL-6, or IFN-γ. Similarly, none of the recombinant cytokines affected the survival of cerebellar neurons, or the survival of the SH-SY5Y neuroblastoma cells. Amyloid-β_1-42 _caused a dose-dependent reduction in the survival of neurons that was not observed after the addition of a control peptide (amyloid-β_42-1_). To determine if cytokines could modify the effects of amyloid-β_1-42_, primary cortical neurons were pre-treated with 1 ng/ml individual cytokines, before the addition of 10 μM amyloid-β_1-42_. There was no significant difference between the survival of neurons pre-treated in control medium and those pre-treated in medium containing TNF-α, IL-1β or IL-6 prior to the addition of amyloid-β_1-42_. In contrast, the survival of neurons pre-treated with IFN-γ and amyloid-β_1-42 _was significantly less than neurons treated with amyloid-β_1-42 _alone (Figure 1). Further studies demonstrated that this effect of IFN-γ was dose-dependent; and a significant reduction in neuronal survival was still observed when cells were treated with 40 pg per ml of IFN-γ (Figure 2).

The effects of IFN-γ were tested on both primary cortical and cerebellar neuronal cultures. Pre-treatment with IFN-γ (100 pg/ml) resulted in reduced survival of both primary cerebellar and cortical neurons following the addition of 10 μM amyloid-β_1-42_. Since it is possible that the effects of IFN-γ in these neuronal cultures were via effects on contaminating astroglial cells, we also tested the effects of IFN-γ on the SH-SY5Y neuroblastoma cell line. Pre-treatment with IFN-γ reduced the survival of SH-SY5Y neuroblastoma cells following the addition of 10 μM amyloid-β_1-42 _indicating that IFN-γ had a direct effect on neuroblastoma cells (Table 1).

**Table 1:** Treatment with IFN-γ reduces neuronal survival following incubation with amyloid-β_1-42_. SH-SY5Y cells or primary neuronal cell cultures were pre-treated with IFN-γ (1 ng/ml) for 24 hours prior to the addition of amyloid-β peptides as shown. Cell survival was determined using the WST-1 test after 24 hours (cell lines) or 4 days (primary neuronal cultures). Each value is the mean percentage cell survival ± SD from triplicate experiments repeated 3 times (9 observations). ** = Neuronal survival significantly less than untreated neurons incubated with amyloid-β_1-42 _(p < 0.05).

	**% Neuronal Survival**			
**Cell Type**	**Amyloid-β_1-42_**		**Amyloid-β_42-1_**	

	**Control**	**IFN-γ**	**Control**	**IFN-γ**

**SH-SY5Y cells**	62 ± 4	33 ± 9**	101 ± 4	98 ± 7
**Cortical neurons**	68 ± 7	36 ± 7**	98 ± 3	96 ± 5
**Cerebellar neurons**	79 ± 4	58 ± 11**	101 ± 8	102 ± 8

To determine if IFN-γ treated neurons show increased sensitivity to other neurotoxins, cortical neurons were treated with 100 pg/ml of IFN-γ prior to exposure to HuPrP82-146, a synthetic correlate of a neurotoxic peptide found in the brains of patients with prion disease [10], staurosporine or hydrogen peroxide. The survival of neurons pre-treated with IFN-γ was significantly less than that of untreated neurons, when incubated with HuPrP82-146. However, there were no significant differences between the survival of neurons treated with IFN-γ and untreated neurons that were exposed to hydrogen peroxide, or to staurosporine, a drug that caused programmed cell death in neurons via activation of the ceramide pathway [11] (Table 2).

**Table 2:** IFN-γ treated neurons show increased sensitivity to HuPrP82-146. Neurons treated with 1 ng/ml IFN-γ for 24 hours prior to the addition of neurotoxins as shown. Cell survival was determined 24 hours later using the WST-1 test. Each value is the mean percentage cell survival ± SD from triplicate experiments repeated 3 times (9 observations). ** = Neuronal survival significantly less than untreated neurons incubated with HuPrP82-146 (p < 0.05).

		**Neuronal survival (%)**	
	**conc**	**Control**	**IFN-γ**

**HuPrP82-146 (μM)**	**40**	48 ± 6	12 ± 7**
	**10**	64 ± 5	22 ± 8**
	**2.5**	92 ± 9	41 ± 9**
**Staurosporine (ng/ml)**	**20**	37 ± 4	40 ± 6
	**5**	78 ± 6	72 ± 8
	**1.25**	92 ± 3	95 ± 4
**Hydrogen peroxide (μM)**	**1**	22 ± 5	18 ± 7
	**0.25**	58 ± 7	55 ± 6
	**0.06**	87 ± 6	89 ± 9

### Caspase-3 activity

**Figure 3:**
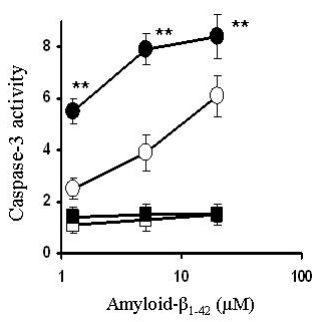
**Pre-treatment with IFN-γ increases amyloid-β_1-42_-induced caspase-3 activity**. Levels of caspase-3 activity in cortical neurons pre-treated with control medium (○), or with 100 ng/ml IFN-γ (●) prior to the addition of varying concentrations of amyloid-β_1-42_. Also shown is the caspase-3 activity from neurons pre-treated with control medium (□), or with 100 ng/ml IFN-γ (■) and incubated with varying concentrations of amyloid-β_42-1_. Values shown are the mean percentage fluorescence units from quadruplicate experiments repeated twice (n = 8), ± SD. ** = Caspase 3 activity significantly greater than untreated neurons incubated with amyloid-β_1-42 _(p < 0.05).

**Figure 4:**
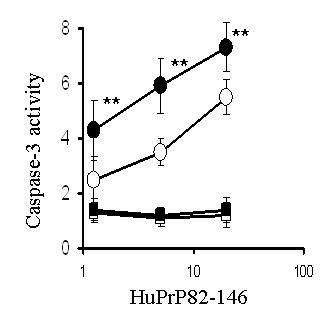
**Pre-treatment with IFN-γ increases HuPrP82-146-induced caspase-3 activity**. Levels of caspase-3 activity in cortical neurons pre-treated with control medium (○), or with 100 ng/ml IFN-γ (●) prior to the addition of varying concentrations of HuPrP82-146. Also shown is the caspase-3 activity from neurons pre-treated with control medium (□), or with 100 ng/ml IFN-γ (■) and incubated with varying concentrations of HuPrP82-146scrambled. Values shown are the mean percentage fluorescence units from quadruplicate experiments repeated twice (n = 8), ± SD. ** = Caspase 3 activity significantly greater than untreated neurons incubated with HuPrP82-146 (p < 0.05).

Caspase-3 is an enzyme that is increased during apoptosis [12] and was measured as an alternative indicator of neuronal injury. Caspase-3 activity was increased in primary cortical neurons treated with amyloid-β_1-42 _or HuPrP82-146, but not in primary cortical neurones treated with control peptides (amyloid-β_42-1 _or HuPrP82-146scrambled) or with IFN-γ (100 pg/ml) alone. Following pre-treatment with 100 pg/ml IFN-γ caspase-3 activity in cortical neurons treated with either amyloid-β_1-42 _or HuPrP82-146 was significantly higher than in untreated cells incubated with amyloid-β_1-42 _or HuPrP82-146 (Figures 3 &4).

### IFN-γ raises cytoplasmic PLA_2 _levels in neurons

**Figure 5:**
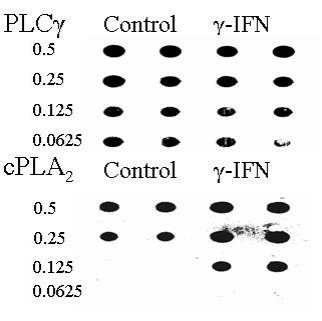
**IFN-γ increases levels of cPLA_2 _in SH-SY5Y neuroblastoma cells**. Immunoblot showing levels of cPLA_2 _and phospholipase C in lysates made from untreated cells and cells that had been treated with 100 pg/ml of IFN-γ for 3 hours. The fractions of the original samples that were added to membrane are shown.

Since recent studies demonstrated that cPLA_2 _is involved in amyloid-β_1-42 _induced neuronal injury [13] we compared levels of cPLA_2 _and another enzyme involved in cell signalling (PLCγ-1) in IFN-γ-treated and untreated SH-SY5Y cells. Treatment with IFN-γ did not significantly alter the total protein content of cells (data not shown). When lysed cells were diluted and analysed in a dot blot we found that SH-SY5Y cells treated with IFN-γ (100 pg/ml) had higher levels of cPLA_2 _than did untreated cells (Figure 5). However, pre-treatment with IFN-γ did not affect the levels of PLCγ-1 indicating that IFN-γ up-regulates specific pathways in these neurons. We next determined if pre-treatment with cytokines affected prostaglandin production. Levels of prostaglandin E_2 _were not altered by any of the cytokines tested. Prostaglandin E_2 _levels were significantly raised after the addition of either amyloid-β_1-42 _or HuPrP82-146, but not after the addition of control peptides (amyloid-β_42-1 _or HuPrP82-146scrambled). Pre-treatment of neurons of 100 pg/ml IFN-γ resulted in increased prostaglandin E_2 _production following the addition of 10 μM amyloid-β_1-42 _or 10 μM HuPrP82-146. Prostaglandin E_2 _levels in neurons incubated with 10 μM amyloid-β_1-42 _or 10 μM HuPrP82-146 was not affected by pre-treatment with TNF-α, IL-1β, IL-6 (Table 3).

**Table 3:** Pre-treatment with IFN-γ increases prostaglandin E_2 _production in response to amyloid-β or HuPrP82-146. SH-SY5Y neuroblastoma cells were pre-treated for 24 hours with 1 ng/ml cytokines as shown prior to the addition of amyloid-β or PrP peptides. Cells were lysed 24 hours later and total prostaglandin E_2 _levels were measured. Each value given represents the mean ± SD from triplicate experiments repeated twice (6 observations). ** = Prostglandin E_2 _levels significantly higher than untreated neurons incubated with amyloid-β_1-42 _(p < 0.05).

	**Prostaglandin E_2 _(pg/ml)**			
	**Amyloid-β_1-42_**	**Amyloid-β_42-1_**	**HuPrP82-146**	**HuPrP82-146sc**

**Control medium**	241 ± 62	< 50	187 ± 40	< 50
**IFN-γ**	443 ± 112**	66 ± 38	303 ± 55**	< 50
**IL-1β**	228 ± 54	< 50	202 ± 46	< 50
**IL-6**	218 ± 66	< 50	210 ± 48	55 ± 35
**TNFα**	255 ± 82	< 50	214 ± 33	< 50

## Discussion

Reports that activated microglial cells are found in close association with damaged neurons in AD raise the possibility that glial-derived cytokines are involved in neuropathogenesis. In the current studies the survival of either primary neuronal cultures (cortical or cerebellar neurons) or SH-SY5Y neuroblastoma cells was not affected by incubation with high concentrations of recombinant cytokines (up to 10 ng/ml). However, while none of the cytokines were directly neurotoxic, pre-treatment with IFN-γ significantly reduced the survival of neurons that incubated with amyloid-β_1-42_. This effect of IFN-γ was dose-dependent and was observed at concentrations previously reported in the cerebral cortex of APP(SWE) transgenic mice [14].

Pre-treatment with IFN-γ also increased the sensitivity of neurons to HuPrP82-146, a neurotoxic peptide found in prion diseases [10]. However, neurons pre-treated with IFN-γ did not demonstrate increased sensitivity to all neurotoxins: there was no change in the neurotoxicity of staurosporine, a drug that causes programmed cell death in neurons via activation of the ceramide pathway [11], or of hydrogen peroxide which causes oxidation of cellular membranes. These observations strengthen the hypothesis that IFN-γ treatment selectively increases the expression of proteins involved in specific apoptotic pathways. Previous reports showed that amyloid-β peptides activate PLA_2 _[15], that PLA_2 _inhibitors protect against the amyloid-β_1-42 _induced neurotoxicity [16], and more specifically, that the cPLA_2 _isoform is required for induced neurotoxicity [13]. The current study showed that IFN-γ increased expression of cPLA_2 _in neurons, a result consistent with previous observations that IFN-γ increases gene expression of cPLA_2 _in epithelial cells [17]. The activation of cPLA_2 _results in the release of arachidonic acid which is subsequently metabolised by the COXs to prostaglandins and in the present study the increased expression of cPLA_2 _in IFN-γ treated neurons was associated with significantly greater amounts of prostaglandin E_2 _produced following the addition of amyloid-β_1-42 _or HuPrP82-146. IFN-γ treatment increased cPLA_2 _levels without affecting levels of PLCγ-1, further evidence that IFN-γ selectively increases expression of specific pathways.

In AD and prion diseases much of the neuronal death occurs though apoptosis [3]. Although neurons incubated with fibrillar PrP/amyloid-β peptides *in vitro *show signs of apoptosis, the precise mechanisms that activate neuronal apoptosis remain unknown. In the present study both amyloid-β_1-42 _and HuPrP82-146 increased neuronal caspase-3 activity, a marker of apoptosis that is increased in AD [18]. IFN-γ has been implicated in the pathogenesis of AD and IFN-responsive mRNAs have been found in Creutzfeldt-Jakob disease [19]. IFN-γ can be produced in the brain by glial cells and IFN-γ immunoreactivity and IFN-γ-gene expression have been detected in human sensory neurons [20]. Thus, these results indicate that IFN-γ has the potential to increase neuronal loss in AD or prion diseases, consistent with a previous report that the induction of IFNs hastens the progression of experimental prion diseases in mice [21].

## Conclusion

We report that pre-treatment with IFN-γ increased the levels of cPLA_2 _in SH-SY5Y neuroblastoma cells without affecting total cellular protein concentrations, or the levels of PLCγ-1. The increased levels of cPLA_2 _were associated with increased prostaglandin E_2 _production in response to amyloid-β_1-42 _or HuPrP82-146. More importantly, pre-treatment with IFN-γ resulted in reduced neuronal survival following the addition of amyloid-β_1-42 _or HuPrP82-146. Such results are consistent with previous observations that cPLA_2 _is involved in neurodegeneration in AD or prion diseases and indicate that IFN-γ may hasten neuronal loss in these diseases.

## Abbreviations

Alzheimer's disease (AD), interferon (IFN), cytoplasmic phospholipase A_2 _(cPLA_2_), phospholipase C (PLC), cyclo-oxygenases (COX), flourometric immunosorbent enzyme assay (FIENA), tumour necrosis factor (TNF), interleukin (IL).

## Competing interests

The author(s) declare that they have no competing interests.

## Authors' contributions

CB was responsible for the conception, planning and performance of experiments, and for writing this manuscript. Both SK and VL prepared western and dot blot analysis. AW contributed to the planning of experiments, interpretation of results and the writing of the manuscript.
